# Hip and Knee Strength Is Not Affected in 12-16 Year Old Adolescents with Patellofemoral Pain - A Cross-Sectional Population-Based Study

**DOI:** 10.1371/journal.pone.0079153

**Published:** 2013-11-13

**Authors:** Camilla Rams Rathleff, William Neill Baird, Jens Lykkegaard Olesen, Ewa Maria Roos, Sten Rasmussen, Michael Skovdal Rathleff

**Affiliations:** 1 Orthopaedic Surgery Research Unit, Aalborg University Hospital, Aalborg, Denmark; 2 Department of Rheumatology, Aalborg University Hospital, Denmark, Aalborg, Denmark; 3 Research Unit for Musculoskeletal Function and Physiotherapy, Institute of Sports Science and Clinical Biomechanics, University of Southern Denmark, Odense, Denmark; 4 HEALTH, Aarhus University, Aarhus, Denmark; The University of Queensland, Australia

## Abstract

**Background:**

One of the rationales behind using strength training in the treatment of adolescents with Patellofemoral Pain (PFP) is that reduced strength of the lower extremity is a risk factor for PFP and a common deficit. This rationale is based on research conducted on adolescents >15 years of age but has never been investigated among young adolescents with PFP.

**Objectives:**

To compare isometric muscle strength of the lower extremity among adolescents with PFP compared to age- and gender-matched pain-free adolescents.

**Methods:**

In 2011 a population-based cohort (APA2011-cohort) consisting of 768 adolescents aged 12–15 years from 8 local schools was formed. In September 2012, all adolescents who reported knee pain in September 2011 were offered a clinical examination if they still had knee pain. From these, 20 adolescents (16 females) were diagnosed with PFP. Pain-free adolescents from the APA2011-cohort (n = 20) were recruited on random basis as age- and gender-matched pairs. Primary outcome was isometric knee extension strength normalized to body weight (%BW) and blinded towards subject information. Secondary outcomes included knee flexion, hip abduction/adduction and hip internal/external rotation strength. Demographic data included Knee Injury and Osteoarthritis Outcome Score (KOOS) and symptom duration.

**Results:**

Adolescents with PFP reported long symptom duration and significantly worse KOOS scores compared to pain-free adolescents. There were no significant differences in isometric knee extension strength (Δ0.3% BW, p = 0.97), isometric knee flexion strength (Δ0.4% BW, p = 0.84) or different measures of hip strength (Δ0.4 to 1.1% BW, p>0.35).

**Conclusion:**

Young symptomatic adolescents with PFP between 12 and 16 years of age did not have decreased isometric muscle strength of the knee and hip. These results question the rationale of targeting strength deficits in the treatment of adolescents with PFP. However, strength training may still be an effective treatment for those individuals with PFP suffering from strength deficits.

## Introduction

Knee pain is common during adolescence and up to 25% of adolescents report having knee pain [1], [2]. One of the most frequent knee conditions among adolescents is Patellofemoral Pain (PFP) [1], [3]. Population-based studies have shown that the prevalence of PFP among adolescents aged 15–19 years of age is between 6 and 7% [1], [3]. These adolescents report long-standing knee pain with average pain duration of more than three years, which suggest that PFP may exist among even younger adolescents. In addition to long standing pain, adolescents with PFP report severe reductions in function and health-related quality of life (HRQoL) [3].

We recently published data showing reduced isometric knee extension strength among adolescents with PFP aged 15–19 years compared to gender and age-matched adolescents without knee pain [4]. On average the adolescents reported knee pain for 3 years. The reduction in knee extension strength was expected as reduced muscle strength is a common deficit among adults (>18 years of age) with PFP [5]. Supporting this, Duvigneaud et al. and Boling et al. discovered that military recruits who later developed PFP had ∼10% lower isokinetic knee extensor and knee flexor peak torque than the recruits who did not develop PFP during basic military training [6], [7]. These results indicate that even small reductions in knee extension and knee flexion strength may constitute a risk factor for developing PFP among adults. Strength deficits is not only located around the knee but also hip abduction, adduction, external and internal rotation strength are reduced [8], [9], [10], [11].

The latest review covering treatment of patients with PFP advocate for strength training as a keystone in the treatment of PFP [12] as strength training offers superior effect compared to no-treatment control [13]. One of the most important rationales behind this treatment is that reduced strength of the lower extremity appears to be both a risk factor and a common deficit. However this is exclusively based on research conducted on adolescents above 15 years of age, adults and military recruits. Adolescents with PFP younger than 15 years of age may be different than adolescents aged 15–19 years because of younger age, a larger proportion being prior to puberty, and shorter pain duration. Furthermore, no randomised trials have been conducted among young adolescents with PFP (<15 years of age) nor have strength deficits been investigated among young adolescents with PFP. Therefor the rationale for treating young adolescents with strength training has never been tested [14], [15].

Given the lack of studies specifically investigating young adolescents with PFP the purposes of this study were to investigate isometric muscle strength around the knee and hip. The primary hypothesis was that adolescents with PFP would have reduced isometric knee extension strength. Secondary hypothesises were that adolescents with PFP would have reduced isometric muscle strength in knee flexion and hip abduction/adduction and hip internal and external rotation.

## Methods

### Study Design

This cross-sectional study compared 20 adolescents diagnosed with PFP to 20 age- and gender matched pain-free adolescents. Both groups were recruited from the same population-based cohort (Adolescent Pain in Aalborg 2011, the APA2011-cohort) [16]. The study was approved by the Ethics committee of North Denmark Region (N-20110020) and the Danish Data Protection Agency. All participants were required to give written informed consent accompanied by their parents’ consent. The study was conducted according to the Declaration of Helsinki. The reporting of the study complies with the ‘Strengthening the Reporting of Observational studies in Epidemiology’ (STROBE) statement [17].

### Recruitment

**Figure 1:**
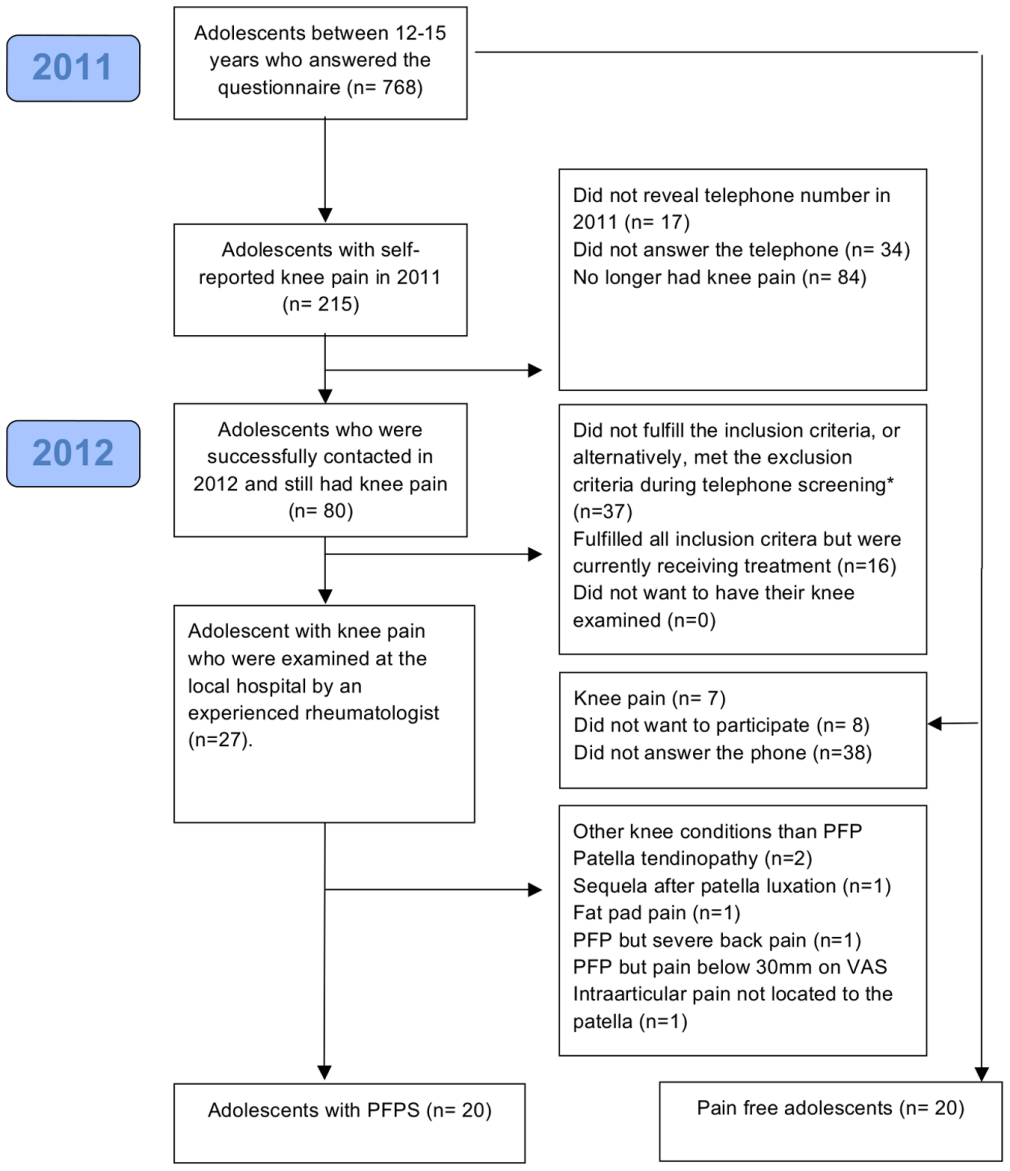
Flow-chart. Flowchart showing recruitment of adolescents with PFPS and gender and age-matched pain free adolescents without knee pain. *Telephone screening: pain for more than 6 weeks; pain felt anteriorly around the patella or diffusely around the knee; no treatment within the previous 12 months, no previous knee surgery and a history of insidious onset of knee pain.

In September 2011 eight lower secondary schools in the community of Aalborg were invited to answer an online questionnaire and to be part of the APA2011-cohort. A total of 768 students aged 12–15 years answered the online questionnaire and 215 students (28%) reported knee pain. In September 2012, all adolescents who reported knee pain in September 2011 were contacted by telephone within a time period of two weeks, Figure 1. Those who still had knee pain were offered a standardised clinical examination by an experienced rheumatologist if they fulfilled the following criteria: pain for more than 6 weeks; pain felt anteriorly around the patella or diffusely around the knee; no treatment within the previous 12 months, no previous knee surgery and a history of insidious onset of knee pain.

The pain-free adolescents were recruited on random basis as age- and gender matched pairs from the same cohort that the adolescents with PFP were recruited from (the APA2011-cohort). Recruitment took place in the same time period as recruitment of adolescents with PFP. The inclusion criteria for the pain-free adolescents were: no current self-reported musculoskeletal pain; no self-reported prior surgery in the lower extremity; no self-reported neurological or medical conditions.

### In- and Exclusion Criteria during the Clinical Examination

During the clinical examination, the students were diagnosed with PFP if they met the following criteria [18]:

- 12–16 years
- Insidious onset of anterior or retro-patellar knee pain for more than 6 weeks and provoked by at least two of the following positions or functions: prolonged sitting or kneeling, squatting, running, hopping or stair walking
- Tenderness on palpation of the patella, or pain with stepping down or double leg squatting; and
- Worst pain experienced during the previous week should be reported to be more than 30 mm on a 100 mm Visual Analogue Scale (VAS).

Exclusion criteria were concomitant injury or pain from the hip, lumbar spine, or other structures of the knee; i.e. tendinopathy or Mb Osgood Schlatter’s disease; previous knee surgery; patellofemoral instability; knee joint effusion; or weekly use of anti-inflammatory drugs. Adolescents who had been treated for PFP using physiotherapy during the last year were excluded. Treatment of PFP would most likely include strength training why inclusion of these adolescents could cause a differential selection bias among adolescents with PFP.

Growing pains (GP) may be mistaken for PFP among young adolescents with PFP. However there are a few important differences between the two pain conditions that was used during clinical examination to distinguish the two pain conditions. Growing Pain is usually non-articular and located to the shins, calves, thighs or popliteal fossa [19]. The pain usually appears late in the day or is nocturnal, often awaking the child. Another distinct difference between the two pain syndromes is the pain debut. Adolescent PFP usually debuts when the adolescent is 11–13 years of age, while GP debut much earlier, at around 6–12 years of age [3], [19].

### Outcome Measurements

Primary outcome were isometric knee extension strength using the best of three consecutive measurements normalized to body weight (%BW). Secondary outcomes included knee flexion, hip abduction/adduction and hip internal/external rotation. Outcome measurements were collected from the most pain-full knee in adolescents with PFP and on an identical proportion of right and left knees among pain-free adolescents. All measurements were done in September 2012 by a rater with previous experience in muscle strength testing. The rater was a physiotherapist and blinded to which of the 40 adolescents were diagnosed with PFP.

### Isometric Muscle Strength

The testing setup included a portable dynamometer and an examination table. Muscle strength was tested with the Mecmesin AFG2500 dynamometer that was attached to the wall through a fixed bolted connection to ensure fixation. All strength tests were isometric strength tests. The test positions were chosen based on procedures that are often applied in clinical settings. To make sure the test procedure was reliable, a pilot study was conducted two weeks prior to the testing of the adolescents (see later header “Reliability”). A total of six movement directions around the knee and hip were tested; knee flexion and extension; hip abduction and adduction; hip internal and external rotation.

During all strength tests the participants were told to stabilize themselves by holding on to the sides of the examination table. A cotton cloth was placed between their lower legs and the strap from the dynamometer to allow for both standardization of the dynamometer placement and pain reduction from the pressure created by the dynamometer.

After receiving instruction about the procedure, the participants were asked to perform one isometric sub-maximal trial. Then an additional practice trial was applied. Afterwards the individual test was administered three times to reduce a possible learning effect. The highest value of three consecutive measurements are presented. A 1-minute rest period was given after each trial. The standardized command given by the examiner was: “Go ahead-push-push-push-push and relax”. To allow for comparison to the latest review covering strength deficits in patients with PFP we reported strength normalized to bodyweight (BW) [5].

**Figure 2:**
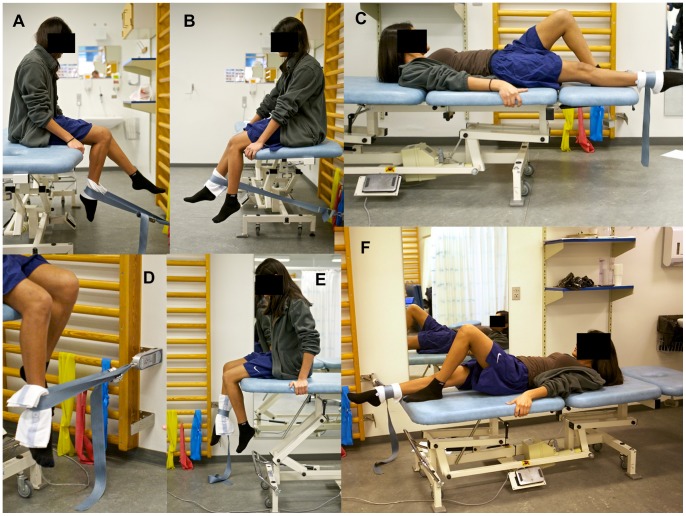
Isometric muscle strength test-positions. The figure shows the test-positions of the six movement directions during isometric knee and hip strength. A: knee flexion, B: knee extension, C: hip abduction, D: hip internal rotation, E: hip external rotation, F: hip adduction.

During knee extension and knee flexion, the strap from the dynamometer was positioned perpendicular to the anterior or posterior aspect of the tibia, 5 cm proximal to the medial malleolus (Figure 2, a & b). Knee extension was tested in a fixed position at 60 degrees of knee flexion while knee flexion was tested during 90 degrees of knee flexion. A pilot study revealed that 90 degrees of knee flexion during knee extension induced knee pain why the adolescents reported they could not exert maximal force due to pain. Therefore 60 degrees of knee flexion was chosen.

During hip abduction and adduction the participant was lying supine on the examination table (Figure 2, C & F). The strap from the dynamometer was positioned perpendicular to the medial or lateral aspect of the tibia, 5 cm proximal to the medial malleolus. The leg was placed in 0 degrees flexion and 0 degrees abduction.

Hip external and internal rotation was tested with the participant sitting on one side of the examination table with the hip and knee flexed at 90 degrees (Figure 2, D & E). The strap from the dynamometer was positioned perpendicular to the lower leg 5 cm proximal to the medial malleolus on either the lateral or medial part of the lower leg. The subject was positioned in an upright position, sitting on the edge of the examination table, with 90 degrees hip and knee flexion.

### Demographics

Secondary outcomes included the patient-reported questionnaire Knee Injury and Osteoarthritis Outcome Score (KOOS) [20] which contains five separate subscales (Pain, Symptoms, Activity in Daily Living (ADL), Function in Sport and Recreation (Sport/Rec), knee-related quality of life (QoL)) that assess the patient’s opinion about their knee and associated problems. This questionnaire was chosen as it has previously been used in young adolescents with knee pain [21]. Further, the Pain Catastrophizing Scale (PCS) was used to assess the participants response to pain [22]. The categories of questions can be divided into: rumination, helplessness and magnification. Health related quality of life was measured by the youth version of the European Quality of Life 5 dimensions (EQ-5D) [23].

Physical activity level was measured with The Physical Activity Scale (PAS) [24]. PAS consists of 9 different activities, where the participant has to fill out 24 hours of work, leisure time and sports on an average weekday. The answers were then transformed into a Metabolic Equivalents (MET). A MET is defined as the oxygen uptake in ml/kg/min with one MET being equal to the oxygen cost of sitting quietly, equivalent to 3.5 ml/kg/min. Age, height, weight, Body Mass Index (BMI) and pain duration were included as demographics.

### Reliability

Before data collection, a test-retest intra-rater reliability study was performed to investigate the reliability and agreement of the isometric strength testing for the best of three measurements. The study included a convenience sample of 17 young adults who were tested twice, with 30 minutes between test and retest. A two-way random effects model (2.1), single measures, absolute agreement, intraclass correlation coefficients (ICC) were used to express intra-rater reliability. Limits of Agreements (LoA) were used to express the agreement between test and retest [25]. Agreement was presented as LoA divided by the mean and multiplied by 100 to represent the maximal difference in percentage in 95% of the measurements (LoA%). ICC for all six movement directions were above>0.92 and LoA% was below 29.2% for all six movement directions. The lowest reliability and agreement was found in hip external rotation.

### Sample Size

The sample-size was based on detecting a difference between groups of at least 20% on the normalized isometric quadriceps strength. This difference was based on quadriceps torque data from our previous study comparing adolescents aged 15–19 years with a similar methodology [4]. Using a common standard deviation of 0.5 Nm/Kg, power of 80%, and an alpha level of 5%, at least 17 adolescents were needed in each group to detect a 20% difference between groups. The number of adolescents diagnosed with PFP one year after inclusion in the APA2011-cohort determined the final sample-size. Therefore 20 adolescents with PFP and 20 pain-free adolescents were included.

### Statistical Analysis

All data were visually inspected using a Q-Q plot. Mean values ±SD are reported if data were normally distributed. If data were non-normally distributed they were presented as median and interquartile range (IQR). Paired samples t-test was used to test the difference in isometric strength between matched pairs. All calculations were performed using Stata version 11 (StataCorp, College Station, Texas, USA).

## Results

**Figure 3:**
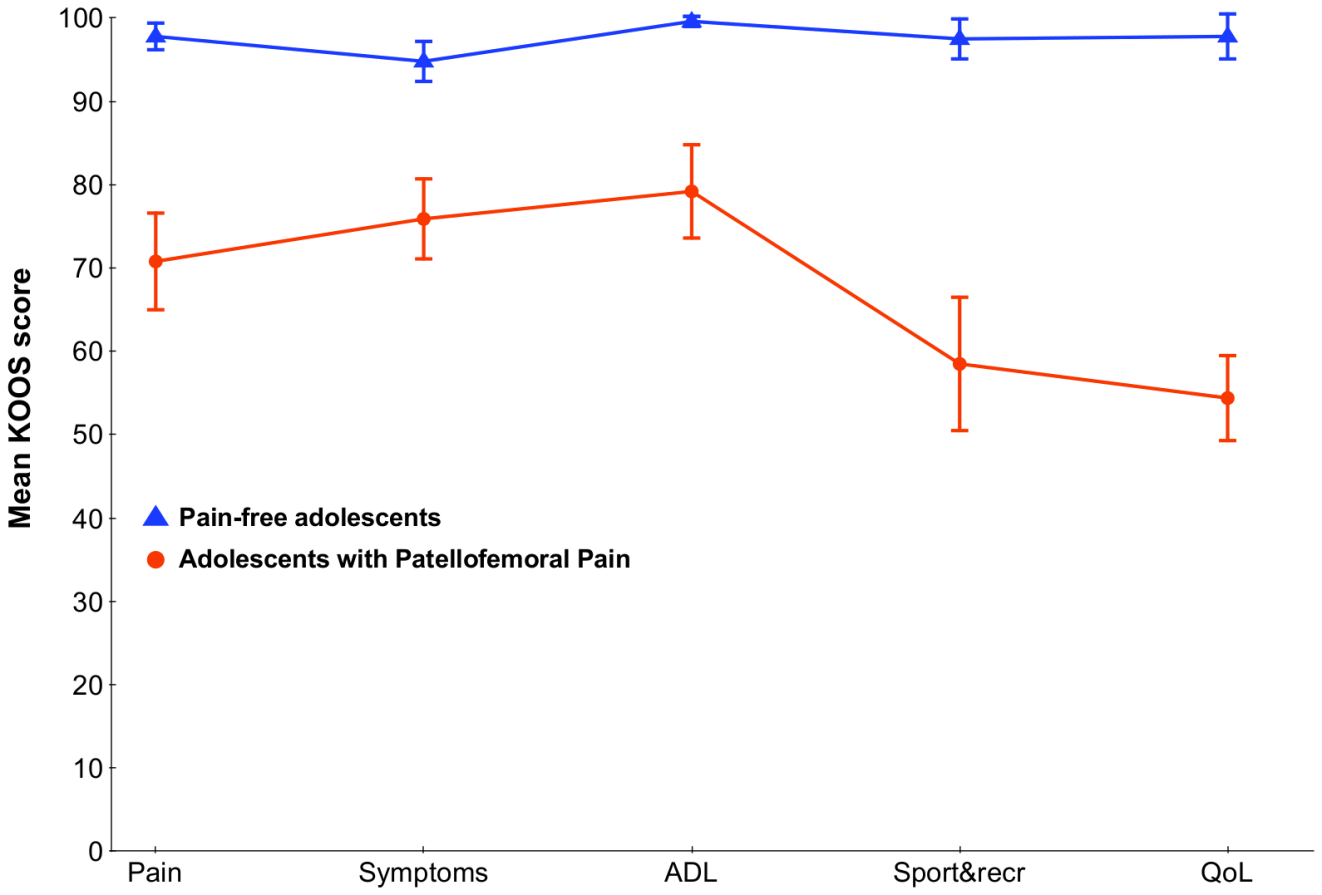
Knee injury and Osteoarthritis Outcome Score: Outcome profile. Mean KOOS subscales are presented and reported as an outcome profile for the adolescents with patellofemoral pain (PFP) versus the age- and gender matched pain-free adolescents. KOOS subscales: Pain, Symptoms, Activity in Daily Living (ADL), Sport and Recreation (Sport&recr) and Knee-related Quality of Life (QoL). Error bars represent 95% confidence intervals.

Average pain duration among adolescents with PFP was 28.5 months (Table 1). Self-reported outcome measures showed higher pain levels and significantly worse KOOS scores across all five domains among adolescents with PFP (Figure 3). On average, the adolescents with PFP had 19–43 points lower KOOS scores. The HRQoL, measured with the EQ-5D youth version, and the participants response to pain measured with the Pain Catastrophysing Scale, were significantly worse among adolescents with PFP compared to pain-free adolescents, Table 2. Physical Activity Scale showed no difference in activity level among adolescents with PFP and pain-free adolescents.

**Table 1:** Demographic data for both groups.

	Adolescents with PFP N = 20	Pain-free adolescents N = 20	p-value
Age [years]	14.6 (1.1)	14.8 (1.0)	0.04*
Height [cm]	167.0 (10.0)	167.4 (8.1)	0.78
Weight [kg]	55.2 (9.0)	56.1 (13.2)	0.61
Gender (number of females)	16	16	1.00
BMI [kg/m^2^]	19.5 (18.2–20.7)**	19.3 (17.4–21.7)**	0.50
Dominant lower extremity (number who replied right leg)	19	19	1.00
Most symptomatic knee (number who replied right knee)	14	n/a	
Pain duration [months]	28.5 (24–36)**		
VAS rest [mm]	5.5 (1–29)**		
VAS worst [mm]	66.5 (52–76.5)**		
VAS activity [mm]	55.5 (40.5–71)**		

* Two adolescents in the pain-free group had their birthday between we called them, and until they were tested. Therefore it appears that the control group is slightly older.

** Presented as median and interquartile range.

Abbreviations: BMI = body mass index. VAS = visual analogue scale.

**Table 2:** Demographic data for both groups*.

	Adolescents withPFP N = 20	Pain-freeadolescents N = 20	Meandifference (95%CI)	p-value
EQ-5D, index score*	0.72 (0.68–0.78)	1.00 (1–1)	−0.23 (−0.28; −0.20)	<0.00001
EQ-5D, visual analog score*	82.5 (72.5–89)	93 (80–99)	−10 (−28; 5)	0.02
PCS score* )	15 (8–23)	5.5 (0–10.5)	7 (4.5;16)	0.003
Physical Activity Scale (Metabolic equivalent)	42.0 (39.4–49.8)	45.1 (41.4–53.0)	−2.7 (−14.4; 4.1)	0.19

Abreviations: EQ-5D: European Quality of Life 5 Dimensions. PAS: Physical Activity Scale. PCS: Pain Catastrophizing Scale (PCS).

* EQ-5D, PAS and PCS are reported as median and interquartile range. Mean differences are presented together with a 95% Confidence Interval (95%CI).

### Primary and Secondary Outcomes

Mean difference between groups in primary outcome, isometric knee extension strength, was 0.3% BW, p = 0.97. The difference in isometric knee flexion strength was 0.4% BW while the differences in hip strength ranged from 0.4 to 1.1% BW, p>0.35 (Table 3). Converting the isometric strength measurements from %BW to torque (Nm) or Nm/kg using leg length or length of the lower leg did not change the results. Likewise, adjusting for age, BMI or physical activity level did not change the magnitude of difference between groups.

**Table 3:** Primary and secondary outcome measures: Isometric muscle strength*.

	Adolescents withPFP Mean (SD)	Pain-free adolescentsMean (SD)	Mean difference(95% CI)	p-value
Knee extension (%BW)	81.5 (20.4)	81.8 (18.3)	−0.3(−13.1; 12.6)	0.97
Knee flexion	32.7 (6.8)	32.3 (6.8)	0.4 (−3.9; 4.7)	0.84
Hip abduction	25.5 (4.4)	24.4 (3.8)	1.1 (−1.3; 3.6)	0.35
Hip adduction	26.3 (6.4)	25.5 (5.0)	0.8 (−3.1; 4.7)	0.68
Hip external rotation	21.1 (4.1)	20.1 (3.8)	1.0 (−1.7; 3.7)	0.44
Hip internal rotation	32.0 (5.8)	32.4 (6.4)	−0.4 (−4.7; 3.9)	0.85

The table show the isometric muscle strength among adolescents with PFP and pain-free adolescents. Strength was normalized to bodyweight (%BW) and best out three trials is reported.

* Data are presented as mean and standard deviation (SD). The mean differences are reported as a 95% Confidence Interval (95% CI) with a corresponding p-value.

## Discussion

This study is the first to compare isometric knee and hip muscle strength among young adolescents with PFP and compare them to age- and gender matched pain-free adolescents from the same population-based cohort. We hypothesized that adolescents with PFP would have significantly lower isometric muscle strength of the knee and hip. Despite self-report of functional limitations and long-lasting severe pain, adolescents with PFP did not have decreased isometric muscle strength of hip and knee compared to age- and gender matched pain-free adolescents.

We have previously reported that adolescents aged 15–19 years showed a significantly lower isometric knee extension strength [4]. However these findings were not reproduced in the current younger cohort, even though the same methodology was used and both study groups were recruited from the APA2011-cohort. On average, the adolescents with PFP between 15 and 19 years of age were 3 years older and reported a 1 year longer symptom duration than the younger adolescents in the current study but reported similar KOOS scores. This could suggest that decreased isometric muscle strength of the knee in adolescents aged 15–19 years may be a consequence of longstanding PFP. We hypothesize that the decreased muscle strength found among 15–19 year olds may be a result of a decreased activity level. Previous studies do indeed suggest that patients with longstanding PFP decrease their activity level [26], [27], [28]. A reduced activity level may not be enough to stimulate the same increases in muscle strength during a period of rapid growth and weight increase as their gender and age-matched peers [29]. Other studies have suggested that PFP may develop after an excessive degree of sports participation and a high activity level [30], [31]. The results showed a trend towards a 3 MET lower physical activity in adolescents with PFP compared to pain-free adolescents. The adolescents with PFP in the current study may already have started to decrease their physical activity level as a consequence of PFP [28], [32], [33]. However the cross-sectional design of the current study does not allow us to infer if adolescents with PFP have altered their physical activity level after they developed PFP.

### Strengths and Limitations

Our sample of adolescents with PFP and the pain-free adolescents were both recruited from the same well-defined population-based cohort. In addition knee pain was confirmed at two time points in the group with PFP, one year apart. The recruitment of a previously untreated population-based sample suggests the results may be generalizable to the broader adolescent population. The few previous studies on adolescents with PFP are all patient-based studies. We recruited adolescents from a closed population-based cohort, which may suggest that the adolescents with PFP have shorter symptom duration and lower pain intensity compared with patients who have already consulted their general practitioner. Only a small number of the adolescents with self-reported knee pain were excluded because they were already receiving treatment (n = 16, 20%) (see figure 1). Worst pain during the previous week and pain duration indicate that our sample of young adolescents with PFP is comparable to patient-based studies with regard to pain levels [10], [15], [34], [35]. The comparison of symptom duration between adolescents in the current study and our previous study of adolescents aged 15–19 years of age should be interpreted with care. Symptom duration may be heavily influenced by recall bias and it is unknown if the knee pain started as part of PFP or was associated with a different knee condition.

The sample-size calculation was based on the results of our previous study [4]. The data used in the sample-size calculation did not hold true for adolescents between 12 and 16 years. The current study could be in risk of being underpowered, but looking at the mean difference between groups (–0.3 to 1.1% of BW), none of the current methods for strength measurements would have been able to significantly detect such a small difference. Also, one could argue that a difference around 1% of BW would not be clinically relevant. In some movement directions, adolescents with PFP had slightly higher isometric muscle strength while in other movement directions it was slightly lower. This difference in directions strengthens the assumption that adolescents with PFP do not have lower isometric muscle strength around the hip and knee compared to pain free adolescents. This study investigated isometric strength around the knee and hip. Other aspects of muscle function such as isokinetic strength and endurance could still be impaired. Furthermore, recent evidence suggests that hip extension may be impaired and future studies should investigate hip extension among young adolescents with PFP.

### Clinical Implications

Different subgroups may respond differently to treatment. The different subgroups of patients with PFP have been discussed earlier but primarily in relation to the efficacy of foot orthoses [36], [37], [38]. Subgrouping is often based on anthropometric characteristics and physical deficits. Based on the results of this study one might speculate if it would also be relevant to subgroup patients with PFP based on age, because the underlying aetiology might be different from that of older adolescents and adults. Based on symptom duration of adolescent PFP in the APA2011-cohort, their knee pain developed when the adolescent was between 11 and 13 years of age [3]. van Linschoten et al reported that almost 70% of their patients had a symptom duration between 2 and 6 months, while the median symptom duration reported by Collins et al. was 28 months with only 25% having symptom duration below 12 months [39], [40]. Even though the stated symptom duration may be influenced by recall bias, they suggest that PFP may develop during adolescence or later in life. This may suggest that adolescent PFP and adult PFP represent two distinct pathologies and should be investigated separately or that adolescent and adult PFP represent the same knee condition at two different stages of disease. Future studies on the development of adolescent PFP and aetiology are highly warranted.

## Conclusion

Despite self-report of functional limitations, long-lasting severe pain and decreased quality of life, 12–16 year old adolescents with Patellofemoral Pain do not have decreased isometric muscle strength of the knee and hip compared to age and gender matched pain-free adolescents. These results question the rationale of targeting strength deficits in the treatment of adolescents with PFP. However, strength training may still be an effective treatment for those individuals with PFP suffering from strength deficits.

## Supporting Information

Checklist S1 **STROBE Checklist.**
(DOCX)
