# Supplementary material for: Hip and Knee Strength Is Not Affected in 12-16 Year Old Adolescents with Patellofemoral Pain - A Cross-Sectional Population-Based Study
Source: PLoS One. 2013 Nov 13;8(11):e79153. doi: 10.1371/journal.pone.0079153 (PMC3827322; doi:10.1371/journal.pone.0079153)
Supplement: Checklist S1 — STROBE Checklist. (DOCX) [file pone.0079153.s001.docx]

STROBE Statement—Checklist of items that should be included in reports of ***cross-sectional studies***

|  | Item No | Recommendation |
| --- | --- | --- |
| **Title and abstract** | 1 | (*a*) Indicate the study’s design with a commonly used term in the title or the abstract. **Done**. |
|  |  | (*b*) Provide in the abstract an informative and balanced summary of what was done and what was found. **Done.** |
| Introduction | | |
| Background/rationale | 2 | Explain the scientific background and rationale for the investigation being reported. **Completed in introduction.** |
| Objectives | 3 | State specific objectives, including any prespecified hypotheses. **Given in the end of the introduction.** |
| Methods | | |
| Study design | 4 | Present key elements of study design early in the paper. **Given in first part of the method section.** |
| Setting | 5 | Describe the setting, locations, and relevant dates, including periods of recruitment, exposure, follow-up, and data collection. **Detailed description given in first part of the method section.** |
| Participants | 6 | (*a*) Give the eligibility criteria, and the sources and methods of selection of participants. Given in the sections “Recruitment” and “In- and exclusion criteria during the clinical examination” |
| Variables | 7 | Clearly define all outcomes, exposures, predictors, potential confounders, and effect modifiers. Give diagnostic criteria, if applicable. **Given in “Outcome measurements.”** |
| Data sources/ measurement | 8* | For each variable of interest, give sources of data and details of methods of assessment (measurement). Describe comparability of assessment methods if there is more than one group. **Given in “Outcome measurements.”** |
| Bias | 9 | Describe any efforts to address potential sources of bias. **Done during the description of recruitment.** |
| Study size | 10 | Explain how the study size was arrived at. **Given in the section “Sample size”** |
| Quantitative variables | 11 | Explain how quantitative variables were handled in the analyses. If applicable, describe which groupings were chosen and why. **Given in “Reliability” and “Statistical analysis”** |
| Statistical methods | 12 | (*a*) Describe all statistical methods, including those used to control for confounding |
|  |  | (*b*) Describe any methods used to examine subgroups and interactions |
|  |  | (*c*) Explain how missing data were addressed |
|  |  | (*d*) If applicable, describe analytical methods taking account of sampling strategy |
|  |  | (*e*) Describe any sensitivity analyses  **Given in “Reliability” and “Statistical analysis”** |
| Results | | |
| Participants | 13* | (a) Report numbers of individuals at each stage of study—eg numbers potentially eligible, examined for eligibility, confirmed eligible, included in the study, completing follow-up, and analysed |
|  |  | (b) Give reasons for non-participation at each stage |
|  |  | (c) Consider use of a flow diagram  **A detailed flow-chart (figure 1) has been provided. Additional details have been given in the method section called “Recruitment”.** |
| Descriptive data | 14* | (a) Give characteristics of study participants (eg demographic, clinical, social) and information on exposures and potential confounders |
|  |  | (b) Indicate number of participants with missing data for each variable of interest  **Given in the result section.** |
| Outcome data | 15* | Report numbers of outcome events or summary measures  **Given in the result section.** |
| Main results | 16 | Give unadjusted estimates and, if applicable, confounder-adjusted estimates and their precision (eg, 95% confidence interval). Make clear which confounders were adjusted for and why they were included  **Given in the result section.** |
|  |  | (*b*) Report category boundaries when continuous variables were categorized. **Not applicable.** |
|  |  | (*c*) If relevant, consider translating estimates of relative risk into absolute risk for a meaningful time period  **Not applicable.** |
| Other analyses | 17 | Report other analyses done—eg analyses of subgroups and interactions, and sensitivity analyses  **Not applicable.** |
| Discussion | | |
| Key results | 18 | Summarise key results with reference to study objectives  **Done in the first few lines of the discussion.** |
| Limitations | 19 | Discuss limitations of the study, taking into account sources of potential bias or imprecision. Discuss both direction and magnitude of any potential bias  **Done in the middle part of the discussion in the section called “Strengths and limitations”.** |
| Interpretation | 20 | Give a cautious overall interpretation of results considering objectives, limitations, multiplicity of analyses, results from similar studies, and other relevant evidence  **Done throughout the discussion.** |
| Generalisability | 21 | Discuss the generalisability (external validity) of the study results  **Done in the discussion.** |
| Other information | | |
| Funding | 22 | Give the source of funding and the role of the funders for the present study and, if applicable, for the original study on which the present article is based  **Completed during submission process.** |
